# Expression of Concern: Imbalanced class distribution and performance evaluation metrics: A systematic review of prediction accuracy for determining model performance in healthcare systems

**DOI:** 10.1371/journal.pdig.0000984

**Published:** 2025-08-08

**Authors:** The PLOS Digital Health Editors

The *PLOS Digital Health* Editors issue this Expression of Concern to inform readers of the following issues that came to light after this article’s [1] publication.

- Two articles cited in [1] and included in the systematic review were retracted due to concerns about potential manipulation of the publication process. Reference 40 [2,3] was retracted before [1] was published, and Reference 49 [4,5] was retracted after [1] was published. A member of the *PLOS Digital Health* Editorial Board advised that the retracted references are not essential to support the research reported in [1].
- The references cited in Table 1 of [1] do not correspond to the description in the table. This issue was not fully resolved in post-publication discussions.
- The References list includes incomplete information for several cited works. These issues were not fully resolved in post-publication discussions.
- The article [1] does not fully comply with PRISMA reporting guidelines or include a completed PRISMA checklist. In post-publication discussions the corresponding author provided a completed PRISMA checklist (S1 File) and the following methodological information, but even with these updates the methodology is not reported in sufficient detail to meet PLOS reporting requirements and enable replication of the literature search:
  - The search strategy used the following sources: PubMed; Google Scholar; Web of Science; Scopus.
  - The search strategy keywords included: predictive modeling in healthcare systems; machine learning prediction accuracy score; disease diagnosis with machine learning; machine learning prediction of disease (chronic kidney, hypertension, breast cancer, machine learning model performance evaluations, fraud detection with machine learning, detection of spam messages with machine learning, machine learning prediction with balanced accuracy score, dealing with class imbalance in machine learning etc.).
  - Searches were restricted to work published in or after 2016.
  - Eligibility Criteria: Study design includes sample population in healthcare settings, interventions with the use of machine learning models and a focus on imbalanced class distribution inequality, evaluation metric outcome such as prediction accuracy score as the determining factor for model performance evaluation in healthcare settings where the incidence of class imbalance is a natural recurring phenomenon.
  - Study Selection Process: Authors (MOA and TF) examined articles selected based on title/abstract screening, full-text review and appropriate method use.
  - Disagreement Resolution Process: Resolution of disagreements involved thorough consultation among all authors.
  - Standardized Data Extraction Process: A standardized data extraction form (S2 File) was used to extract relevant study details including research type, methodology, evaluation metric used and score value obtained.
  - Risk of Bias: This study reports on synthesis-level risk of bias arising from availability of selective publications involving machine learning with class imbalance datasets from selective reporting on evaluation metrics.
  - Limitations and Implications: In this review, emphasis on prediction accuracy score use as the determining factor for best model estimation is assessed in the context of healthcare and other real-world application settings where class imbalance distribution datasets exist. The proposed model for performance evaluation is applicable within the stated context. Implications for this review emphasized the use of the proposed model in settings where false alarms pose a greater challenge due to the negative effect it has on larger segments of the population.

## Supporting information

S1 File PRISMA checklist. (DOCX)

S2 File Standardized data extraction form. (DOCX)
