# Supplementary material for: Imbalanced class distribution and performance evaluation metrics: A systematic review of prediction accuracy for determining model performance in healthcare systems
Source: PLOS Digit Health. 2023 Nov 30;2(11):e0000290. doi: 10.1371/journal.pdig.0000290 (PMC10688675; doi:10.1371/journal.pdig.0000290)
Supplement: S1 PRISMA Checklist — PRISMA checklist indicates processes used in Identifying, screening with inclusion and exclusion criteria of related works evaluated in this study. (PDF) [file pdig.0000290.s001.pdf]

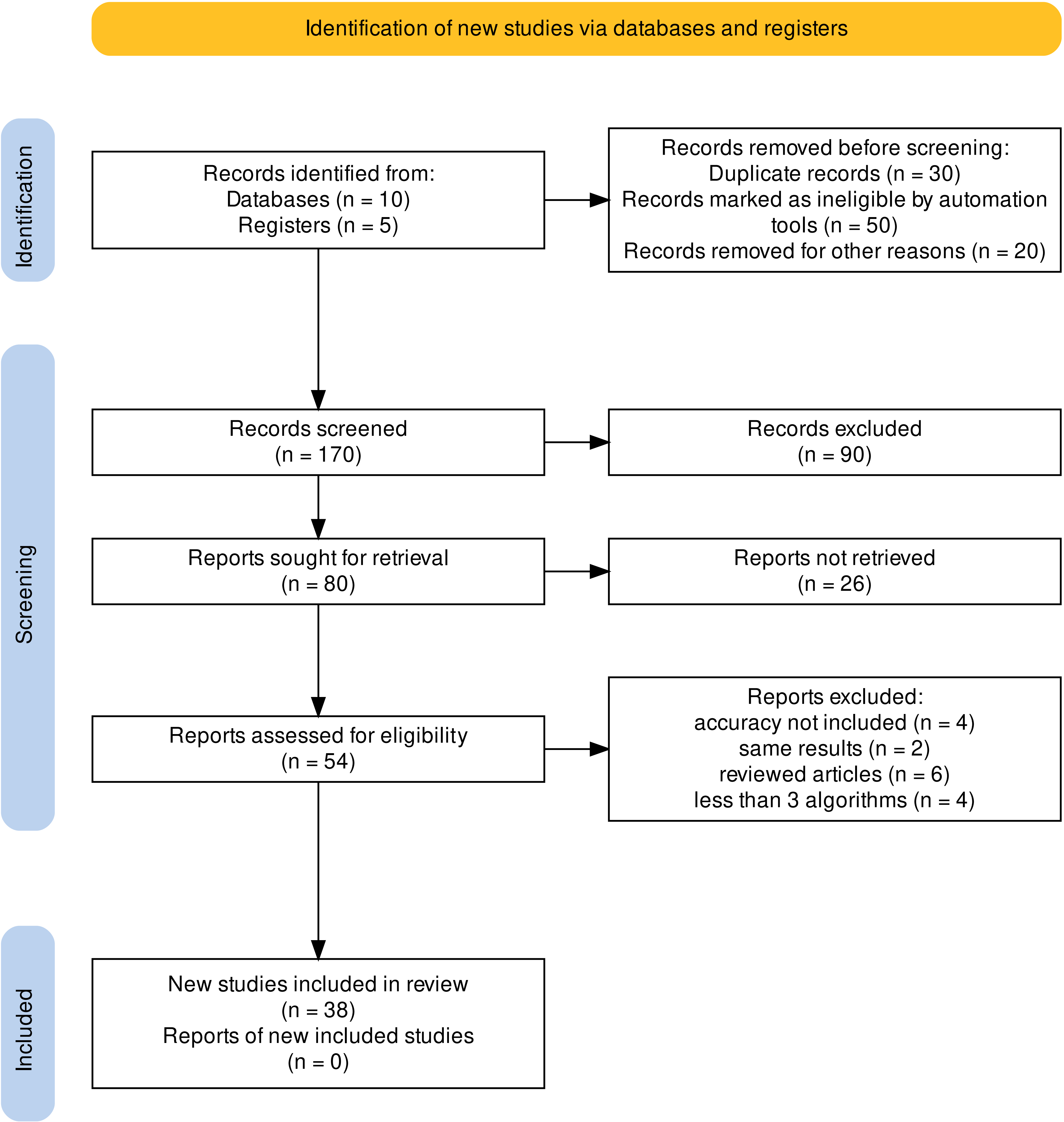
Identification of new studies via databases and registers
Identification
Screening
Included
Records identified from:
Databases (n = 10)
Registers (n = 5)
Records removed before screening:
Duplicate records (n = 30)
Records marked as ineligible by automation tools (n = 50)
Records removed for other reasons (n = 20)
Records screened
(n = 170)
Records excluded
(n = 90)
Reports sought for retrieval
(n = 80)
Reports not retrieved
(n = 26)
Reports assessed for eligibility
(n = 54)
Reports excluded:
accuracy not included (n = 4)
same results (n = 2)
reviewed articles (n = 6)
less than 3 algorithms (n = 4)
New studies included in review
(n = 38)
Reports of new included studies
(n = 0)
