# Supplementary material for: Imbalanced class distribution and performance evaluation metrics: A systematic review of prediction accuracy for determining model performance in healthcare systems
Source: PLOS Digit Health. 2023 Nov 30;2(11):e0000290. doi: 10.1371/journal.pdig.0000290 (PMC10688675; doi:10.1371/journal.pdig.0000290)
Supplement: Attachment — Submitted filename: Reviewer comment_response_plos_one.docx [file pdig.0000290.s002.docx]

Dear Sir,

We would like to express our sincere gratitude to you for the opportunity given to submit a revised draft of our manuscript initially titled *‘A systematic review of prediction accuracy as an evaluation measure for determining machine learning model performance in healthcare systems’* which is now titled ‘***Imbalanced class distribution and performance evaluation metrics, a systematic review of prediction accuracy for determining model performance in healthcare systems***’ as requested to reflect research review focus.

We appreciate efforts spent by yourself and the reviewers in providing such an invaluable feedback.We’re happy to report that reviewer comments from the feedback have largely been addressed and incorporated into the revised manuscript. Where necessary, others have been deleted or modified to meet required criteria.

Response to reviewer comments and concerns are listed below for emphasis.

**Reviewer Comments**

Reviewer#1: Dear Author,

Comment: *1*. *The title of manuscript needs re-synthesis as in its present form it is not acceptable.*

**Response**: Thank you for the direction. We have made changes to the title to emphasize research focus

Comment: *2. The methodilogy is vague and too many subtopics in each section of manuscript compromises logical flow of manuscript.*

**Response**: Thank you for the comment, we followed PRISMA & 2020 guidelines specified for systematic review hence the many subtopics under methodology to show specific activities performed.

Comment *3. Subheading Result part is missing and reults are aglomerated in methodology.*

**Response:** Thank you for the comment, we have created a new section **3.0 Results** to show balanced accuracy process diagram indicating how it is achieved together with a flowchart of PRISMA process flowchart.

Comment 4. *The manuscript need to be edited rigorously by native English speaker for English language and grammar*

**Response:** As much as possible a thorough review of all sentences has been made to improve on English language used. Duplicate sentence has been removed and many other errors corrected.

**Reviewer #2:** *There is no universal method that can be considered superior across all diverse datasets. Different methods cannot be directly compared across varying datasets, even when using the same methodology. While formal meta-analyses like the G-test might not exist, it's important to note that comparisons are complex. Lastly, it's worth highlighting that calibrating a model cannot solely rely on AUC or confusion matrices – there are various other approaches to consider.*

**Response:** it is indeed true that no universal method considered superior across all diverse datasets exists. Our performance calibration has been emphasized now clearly to be based on dataset with class distribution inequality as it pertains to real-world applications such as healthcare systems and other non-medical settings such as fraud detection in bank transactions. Inherent in balanced accuracy score determination are model estimation of true positive rates, false positives, true negatives and false negatives.

**Reviewer #3**: *The research findings has been well presented based on the available literature related to the ML algorithms and their advancement in the field of Medical Sciences. With the advancement in AI and ML based medical technologies, proper assessment of the methods is required.*

**Response**: Thank you for the compliments. It is our firm belief that model performance evaluation on imbalanced dataset with class distribution inequality specially found in real-world applications such as healthcare systems may benefit from using balanced accuracy score to determine model performance as opposed to prediction accuracy score.

**Reviewer #4: Summary**
*1*.        *The Research article addresses a very modern aspect in the use of digital tools in health.
2.        The methodology is fairly broken down in details with the exact methods stated and elaborated such as the PRISMA protocol which is commonly used for systematic reviews. The inclusion and exclusion criteria of the paper selections are clear enough*.

3.        *The Machine learning knowledge elaborated and expressed in the paper is good and diverse exploring some rarely used metrics such as balanced accuracy.
4.        The conclusion is supported by the data and the results presented.*

**Response:** Thank you for the compliments.

**Minor issues:**

1. *I would have liked to see research questions or the specific objectives stated or listed together independently in the related works summary as they are not easily noted when spread in the the literature of the related works.*

**Response:** Thank you for the direction, sub-sections to indicate research questions and research objectives have been created to emphasize more clearly, research questions and research objectives.

2. *Briefly at the categorization of your research as either qualitative or quantitative or even both. From observation, I see some substantial amounts of quantitative methods*.

**Response:** Yes, our research approach combines qualitative and quantitative techniques as shown in the number of research materials obtained and used due to some criteria.

*3.   The method of specificity was assigned to sensitivity just below the Receiver operating characteristic curve (ROC).    Sensitivity = TN / (TN + FP) it should have been specificity equated to that method.*

**Response**: Thank you for the correction, please pardon us for this omission which has been corrected.

4. *It would be better to have the method of precision and fl score elaborated just like the others to help the readers put into perspective how they relate to the confusion matrix.*

**Response**: Thank you. This has been addressed with brief description of f1-score application use and formula for achieving both f1-score and precision.

5.        *Maintain the same units of percentages % in the section above the “works related Summary” section. I suggest you replace the decimals like 0.98 to percentages for consistence?*

**Response**: This has been addressed for all such instances.

5. *In the discussion section, in this phrase “(false alarms) within the healthcare system will be more beneficial to estimate best model performance than a focus on prediction accuracy score.” I suggest to replace will be with could be as I feel there is no sufficient evidence in this paper to justify the claim. The false alarms could have different impact once used on different datasets and using different ML models in comparison with the prediction accuracy*.

**Response**: Thank you for the direction, we have re-phrased that sentence to reflect this important opinion which is crucial in scientific writing.

7.        *In the “Evaluation metrics in Classification” section: “Additional studies on predictive modeling of chronic diseases such as sclerosis progression and outcomes using ML techniques [35] using ML techniques such as K-nearest neighbor”, what were the scores of prediction accuracy of the respective modules in that study. Also typo in repetition of the phrase “ML techniques*”

**Response**: Acknowledged and addressed. Accuracy scores for two periods (6 years and 10 years) have been included.

*8.        There are some typos and grammar issues in the article which will need to be worked on such as:
a)        Some very Long sentences in the introduction section.
b)        In the section for “recall”, maintain the same font size, etc*

**Response**: We are grateful for drawing attention to this ommission. Many have largely been addressed.

Additional Clarifications

Sections have been re-aligned in response to some reviewer comments, main topic has been modified to emphasize focus. We stand ready to address any further concerns that may come up and look forward to hearing from you in due course with respect to our submission.

Yours Sincerely,

Signed

Michael Owusu-Adjei (corresponding author email: mowusuajdie@st.knust.edu.gh)
