# Supplementary material for: Imbalanced class distribution and performance evaluation metrics: A systematic review of prediction accuracy for determining model performance in healthcare systems
Source: PLOS Digit Health. 2023 Nov 30;2(11):e0000290. doi: 10.1371/journal.pdig.0000290 (PMC10688675; doi:10.1371/journal.pdig.0000290)
Supplement: Attachment — Submitted filename: Reviewer comment_response_plos_one_2.docx [file pdig.0000290.s003.docx]

Dear Sir,

We would like to express our sincere gratitude to you for the opportunity given to submit a revised draft of our manuscript initially titled *‘A systematic review of prediction accuracy as an evaluation measure for determining machine learning model performance in healthcare systems’* which is now titled ‘***Imbalanced class distribution and performance evaluation metrics, a systematic review of prediction accuracy for determining model performance in healthcare systems***’ as requested to reflect research review focus.

We appreciate efforts spent by yourself and the reviewers in providing such an invaluable feedback. We’re happy to report that reviewer comments from the feedback have largely been addressed and incorporated into the revised manuscript. Where necessary, others have been deleted or modified to meet required criteria.

Response to reviewer comments and concerns are listed below for emphasis.

**Reviewer Comments**

Comment: *2. In Introduction Section: Replace "patient compliance" with "patient response".*

**Response**: Thank you for the comment. Patient compliance is now replaced with patient response

Comment: *3. Introduction Section: Give examples for usage of predictive modeling performance in current research works with appropriate references.*

**Response**: Thank you for the comment, related studies that assessed predictive model performance in healthcare systems have been included in the introduction section

Comment 4. *Section 1.1.0 ( Accuracy score in non-health settings) - This section doesn't provide info on whether examples mentioned in the section consist of datasets with unequal class distributions or not?.*

**Response:** Thank you for the comment, studies cited in this section are real-world applications with dataset class imbalance characteristics cited to indicate the prevalence of dataset class imbalance in both healthcare systems and other non-health settings.

Additional Clarifications

We stand ready to address any further concerns that may come up and look forward to hearing from you in due course with respect to our submission.

Yours Sincerely,

Signed

Michael Owusu-Adjei (corresponding author email: mowusuajdie@st.knust.edu.gh)
