# Supplementary material for: Expression of Concern: Imbalanced class distribution and performance evaluation metrics: A systematic review of prediction accuracy for determining model performance in healthcare systems
Source: PLOS Digit Health. 2025 Aug 8;4(8):e0000984. doi: 10.1371/journal.pdig.0000984 (PMC12333973; doi:10.1371/journal.pdig.0000984)
Supplement: S1 File — (DOCX) [file pdig.0000984.s001.docx]

| **Section and Topic**  PRISMA CHECKLIST | **Item #** | **Checklist Item** | **Page #** | **Response from Manuscript** |
| --- | --- | --- | --- | --- |
| **TITLE** | 1 | Identify the report as a systematic review, meta-analysis, or both. | 1 | The title states: “Imbalanced class distribution and performance evaluation metrics: A systematic review of prediction accuracy for determining model performance in healthcare systems.” |
| **ABSTRACT** | 2 | Provide a structured summary (background, objectives, methods, results, limitations and conclusions. | 1 | Abstract provides a backgroung to class imbalance distributions in real-world applications and mentions the use of prediction accuracy as the determining factor for model performance in class imbalance distribution datasets. |
| **INTRODUCTION** |  |  |  |  |
| Rationale | 3 | Describe the rationale for the review in the context of existing knowledge. | 1–2 | Introduction discusses the importance of predictive modeling in healthcare, the prevalence of imbalanced datasets in real-world applications such as healthcare systems, and the limitations of using prediction accuracy as the sole metric for determining best model performance. |
| Objectives | 4 | Provide an explicit statement of the objective(s) or question(s) the review addresses. | 2 | Objective: To appraise the effects of using prediction accuracy versus balanced accuracy in imbalanced healthcare datasets. |
| **METHODS** |  |  |  |  |
| Eligibility criteria | 5 | Specify the inclusion and exclusion criteria for the review and how studies were grouped. | 2 | Criteria for both inclusion and exclusion by specify study design, sample population in healthcare settings, intervensions with the use of machine learning models and a focus on imbalanced class distribution inequality. These criterions also specify emphasis on evaluation metric outcome such as prediction accuracy score as the determining factor for model performance evaluation in healthcare settings where the incidence of class imbalance is a natural recurring phenomenon. |
| Information sources | 6 | Specify all databases, registers, websites, reference lists, and other sources searched. | 2 | Search strategy use the following sources for materials that included publications in widely accepted and highly rated journals such as; PuMed, Google scholar, Web of science indexed journals, Scopus indexed journals, IEEE Xplore, Springer nature, Hindawi, Elsevier, Science Direct, IEEEAccess, Plos one) and many others. |
| Search strategy | 7 | Present the full search strategies for all sources, including filters and limits used. | 2 | Search filters included limits on time periods such as publication dates. Publications considered were determined from 2016 to ensure access to most materials since ML use in healthcare has been limited since its inception. |
| Selection process | 8 | Specify methods used to decide study inclusion, number of reviewers, independence, automation. | 2 | Study Selection Process included all authors with vast expreience in systematic review processes in academic writing who examined articles selected based on title/abstract screening, full-text review and appropriate method use. |
| Data collection process | 9 | Specify methods used to collect data, number of reviewers, independence, automation. | 2 |  |
| Data items | 10 | List and define all outcomes for which data were sought. Describe assumptions made. | 2–6 | A standardized data extraction form to extract relevant study from studies included specified study characteristics such as research type, methodology, evaluation metric used and score value obtained. |
| Study risk of bias assessment | 11 | Specify methods used to assess risk of bias, tools used, reviewer numbers, independence, automation. | 2 | Risk of Bias reports on synthesis-level risk of bias arising from availability of selective publications involving machine learning with class imbalance datasets from selective reporting on evaluation metrics |
| Effect measures | 12 | Specify for each outcome the effect measure(s) used in the synthesis or presentation of results. | 2–6 | Research type, methodology, evaluation metric used and score value obtained |
| Synthesis methods | 13 | Describe processes used to decide which studies were eligible for each synthesis. | 2 | Quantitative systhesis approach due to the application of machine learning techniques. |
| **RESULTS** |  |  |  |  |
| Study selection | 16 | Describe the results of the search and selection process, ideally using a flow diagram. | 2 | Flow diagram provided in a PRISMA file named PRISMA_FLOWCHART |
| Study characteristics | 17 | Cite each included study and present its characteristics. | 2–6 | Many studies cited in “Related works” section, with brief summaries of ML techniques and accuracy results. Summary table of characteristics is shown in Table 1 |
| Risk of bias in studies | 18 | Present assessments of risk of bias for each included study. | 2–6 | Risk of Bias reports on synthesis-level risk of bias |
| Results of individual studies | 19 | For all outcomes, present summary statistics and effect estimates for each study. | 2–6 | Individual studies summarized, Research type, methodology, evaluation metric used and score value reported. |
| **DISCUSSION** |  |  |  |  |
| Discussion | 23 | General interpretation, limitations, implications for practice, policy, and future research. | 6–7 | Discusses importance of considering balanced accuracy, limitations of prediction accuracy, and implications for model selection in healthcare. |
| **OTHER INFORMATION** |  |  |  |  |
| Support | 25 | Describe sources of financial or non-financial support and funder roles. | 1 | No funding support was received |
| Competing interests | 26 | Declare any competing interests of review authors. | 1 | Authors report no competing interests |
| Availability of data, code, and other materials | 27 | Report which materials are publicly available and where. | 1 | Available in references |

**Summary of Key Sections:**

- **Abstract:** Reviews the use of prediction accuracy in ML for healthcare, highlights the issue of class imbalance, and suggests other evaluation metrics such as balanced accuracy as a better metric.
- **Introduction:** Explains the importance of predictive modeling, the challenge of imbalanced datasets, and sets the objective to compare prediction accuracy and balanced accuracy.
- **Methods:** Explicitly defines eligibility criteria, search strategy, or risk of bias assessment.
- **Results:** Summarizes findings from various studies using different ML techniques and performance metrics, mainly on prediction accuracy score.
- **Discussion:** Emphasizes the need for appropriate metrics in imbalanced datasets especially in real-world application contexts such as healthcare systems.
