# Supplementary material for: Expression of Concern: Imbalanced class distribution and performance evaluation metrics: A systematic review of prediction accuracy for determining model performance in healthcare systems
Source: PLOS Digit Health. 2025 Aug 8;4(8):e0000984. doi: 10.1371/journal.pdig.0000984 (PMC12333973; doi:10.1371/journal.pdig.0000984)
Supplement: S2 File — (DOCX) [file pdig.0000984.s002.docx]

Data collection form

| **Study ID** |
| --- |
|  |

**Review title**

| 1. **Date form completed**  ***(dd/mm/yyyy)*** |  |
| --- | --- |
| 2. **Name of person extracting data** |  |
| 3. **Reference details** |  |

1. **Research Type**

|  | **Description** |  |
| --- | --- | --- |
| 4. **Type of research** |  |  |

1. **Methodology**

|  | **Descriptions as stated in report/paper** | **Location in text** |
| --- | --- | --- |
| 5. **Study topic** |  |  |

1. **Models used as Evaluation metrics**

|  | **Description as stated in report/paper** | **Location in text** |
| --- | --- | --- |
| 6. **ML techniques used** |  |  |

1. **Performance score recorded**

|  | **Description as stated in report/paper** | **Location in text** |
| --- | --- | --- |
| 7. **Model score recorded** |  |  |
